# Umbilical cord mesenchymal stem cells for regenerative treatment of intervertebral disc degeneration

**DOI:** 10.3389/fcell.2023.1215698

**Published:** 2023-08-03

**Authors:** Huagui Huang, Xin Liu, Jinzuo Wang, Moran Suo, Jing Zhang, Tianze Sun, Wentao Zhang, Zhonghai Li

**Affiliations:** ^1^ Department of Orthopedics, First Affiliated Hospital of Dalian Medical University, Dalian, China; ^2^ Key Laboratory of Molecular Mechanism for Repair and Remodeling of Orthopedic Diseases, Dalian, Liaoning, China

**Keywords:** intervertebral disc, intervertebral disc degeneration, mesenchymal stem cells, umbilical cord, tissue engineering

## Abstract

Intervertebral disc degeneration is thought to be a major contributor to low back pain, the etiology of which is complex and not yet fully understood. To compensate for the lack of drug and surgical treatment, mesenchymal stem cells have been proposed for regenerative treatment of intervertebral discs in recent years, and encouraging results have been achieved in related trials. Mesenchymal stem cells can be derived from different parts of the body, among which mesenchymal stem cells isolated from the fetal umbilical cord have excellent performance in terms of difficulty of acquisition, differentiation potential, immunogenicity and ethical risk. This makes it possible for umbilical cord derived mesenchymal stem cells to replace the most widely used bone marrow-derived and adipose tissue derived mesenchymal stem cells as the first choice for regenerating intervertebral discs. However, the survival of umbilical cord mesenchymal stem cells within the intervertebral disc is a major factor affecting their regenerative capacity. In recent years biomaterial scaffolds in tissue engineering have aided the survival of umbilical cord mesenchymal stem cells by mimicking the natural extracellular matrix. This seems to provide a new idea for the application of umbilical cord mesenchymal stem cells. This article reviews the structure of the intervertebral disc, disc degeneration, and the strengths and weaknesses of common treatment methods. We focus on the cell source, cell characteristics, mechanism of action and related experiments to summarize the umbilical cord mesenchymal stem cells and explore the feasibility of tissue engineering technology of umbilical cord mesenchymal stem cells. Hoping to provide new ideas for the treatment of disc degeneration.

## 1 Introduction

With the growth of the global population and accelerated aging, low back pain (LBP) has become common public health problem worldwide (Cieza et al., 2021). Currently, the prevalence of LBP is 9.4% worldwide and is the leading cause of disability and loss of productivity (Geurts et al., 2018; Wu et al., 2020; Knezevic et al., 2021). Annually, the direct cost of treating LBP is approximately US$ 30 billion and the indirect socio-economic losses account for approximately US$ 100 billion in the United States (Binch et al., 2021). Although the causative factors of LBP are complex and varied, intervertebral disc degeneration (IDD) is considered to be the most common cause of LBP(Maher et al., 2017). IDD can be caused by cellular senescence, genetics, mechanical loading, obesity and even smoking (Adams and Roughley, 2006; Xin et al., 2022).

At present, the treatment of IDD mainly includes conservative treatment and surgical treatment. Although these treatments can provide good relief to patients, they do not slow or reverse the reduction of extracellular matrix (ECM) and nucleus pulposus cells (NPCs), do not repair the patient’s disc tissue, and have unsatisfactory long-term outcomes. In contrast, regenerative therapies based on restoring the physiological structure and biomechanical function of the intervertebral disc (IVD) have gained widespread interest in recent years. Among them, the current research related to mesenchymal stem cells (MSCs) for IDD is the most extensive. MSCs are pluripotent stem cells with the ability to differentiate into a tri-spectrum of osteoblasts, chondrocytes and adipocytes (Pittenger et al., 1999). Positivity for surface markers CD73, CD105 and CD90 is a distinctive feature (Dominici et al., 2006). MSCs are used as the first choice for regenerating IVD because of their abundant source and easy availability, extremely low immunogenicity, strong ability to induce differentiation, and to proliferate in a low-oxygen, low-sugar environment (Miao et al., 2006).

In the past, bone marrow mesenchymal stem cells (BMMSCs) were considered to be the gold standard of regenerative therapy. However, recent studies have found that umbilical cord mesenchymal stem cells (UCMSCs) are superior to BMMSCs in terms of cell source and differentiation potential. The IVD is the largest avascular tissue in the body (Molladavoodi et al., 2020). The survival of UCMSCs injected into the intervertebral disc is limited by the complex anatomy and harsh microenvironment within the disc (Sun et al., 2020). In recent years, with the continuous development of biomaterials with good biocompatibility as well as mechanical properties, tissue engineering techniques by implanting pretreated MSCs into biomaterial scaffolds have compensated for the shortcomings of MSCs for the treatment of IDD. However, there is still controversy regarding the selection of the most suitable cell type as well as the biological material. In this paper, we explored the effectiveness and feasibility of UCMSCs and their tissue engineering for regenerating IVD, and we hope that this study will help researchers to explore more systematically the prospects of UCMSCs in regenerating IVD.

## 2 IVD and IDD

IVD is the largest avascular tissue in the body and consists of fibrocartilage tissue, which is one of the most important structures of the spine. Anatomically, IVD consists of three main components: the central highly hydrated gelatinous nucleus pulposus (NP), fibrous rings (AF) consisting of thin sheets of collagen fibers around the NP, and cartilage end plate (CEP) is a transparent cartilage structure that connects adjacent vertebrae (Smith et al., 2011; Molladavoodi et al., 2020). The above structure distributes the axial load from the spinal cone and increases the mobility of the spine. NP is rich in proteoglycans and type II collagen, and it is highly hydrated, such that physiologic osmotic pressures readily dissipate any mechanical forces transmitted through the spine (Frith et al., 2013). AF is a laminar structure consisting mainly of type I collagen in a highly oriented manner, with approximately 15–25 layers. The closer the AF is to NP, the higher content of type II collagen and water (Torre et al., 2019; Kamali et al., 2021). CEP consists of hyaline cartilage that lies between the soft tissue of the IVD and the bony structures of the vertebral body. CEP is essential to maintain the mechanical integrity of the IVD and the exchange of nutrients (Zhang X. B. et al., 2021). Essential substances such as glucose and oxygen permeate into the NP mainly through the CEP to maintain IVD activity (Frost et al., 2019). ECM is present in the extracellular environment of all tissues. The main components of the ECM within the IVD include: collagens, proteoglycan, and non-collagenous proteins. The intensity of normal IVD is primarily affected by the components of ECM (Roughley et al., 2006; Liang et al., 2022).

The specific mechanism leading to IDD is still unclear, and the prevailing view is that cell senescence, inadequate nutritional supply, repeated mechanical stress, obesity, trauma, genetics, and even smoking can lead to the development of IDD (Adams and Roughley, 2006; Gübitz et al., 2018; Okada et al., 2019; Zhang et al., 2019). Among them, genetic factors are the main cause of IDD, approximately 50%–70% of the variability in disc degeneration is caused by an individual’s genetic inheritance (Battié et al., 1995; Buckwalter, 1995; Battié et al., 2008). In the early stages of IDD, there is an altered NPCs phenotype and a decrease in cell numbers, as well as an upregulation of the expression of enzymes such as MMP that mediate the degradation of ECM. This results in the inhibition of proteoglycans, glycosaminoglycans, aggrecan and type II collagen production, and an increase in type I collagen production. Ultimately, this leads to a weakening of IVD hydration and a reduction in height. The axial pressure from the spine is dispersed through the NP to the adjacent AF, which leads to altered biomechanical function of the AF as well as structural damage (Freemont, 2009; Morris et al., 2021; Ohnishi et al., 2022). The rupture of AF provides a suitable microenvironment for the growth of sensory neurons and blood vessels, prompting their growth into the IVD and accelerating the development of pain (Yee et al., 2016). As IDD progresses further, high levels of inflammatory cytokines are produced by AF cells, NPCs, and immune cells. These inflammatory factors, such as interleukin (IL)-1β and tumor necrosis factor (TNF)-α, further aggravate IDD by exacerbating the inflammatory response, inhibiting IVD cell proliferation and differentiation, accelerating cellular senescence and apoptosis, and promoting ECM degradation (Rogers et al., 2017; Song et al., 2017; Wang et al., 2020). Calcification of CEP exacerbates metabolic disturbances within the IVD by affecting the exchange of substances (Wang et al., 2018). In addition, the above-mentioned series of pathological changes promote the production of reactive oxygen species (ROS) within the IVD and form a positive feedback loop, accelerating apoptosis within the disc (Feng et al., 2017).

## 3 Traditional treatment for IDD

Currently, the traditional treatment modalities for IDD include conservative treatment and surgery, and it is critical to choose the appropriate treatment for patients with different degrees of degeneration and clinical manifestations. For patients with early IDD, pharmacotherapy has a clear role in controlling pain and improving patient function and quality of life (van Middelkoop et al., 2011). However, the long-term use of drugs may lead to serious side effects as well as potential drug addiction and dependence, which makes the clinical use of drug therapy controversial (Hale et al., 2005; Wong et al., 2017). In addition, non-pharmacological treatments such as bed rest, brace immobilization, acupuncture, massage, electromagnetic or electrothermal therapy are also very effective in relieving acute attacks of LBP, and they are often combined with pharmacological treatments and surgery (Wegner et al., 2013; Glazov et al., 2014).

For patients with advanced IDD and those who have failed conservative treatment, surgery becomes the best option. Common surgical procedures include spinal decompression, spinal fusion and total disc replacement. Currently, spinal fusion is the most widely used in clinical practice and is considered the gold standard for the surgical treatment of IDD (Lee et al., 2016). Spinal fusion improves the stability of the spine and reduces patient pain by joining and fusing adjacent vertebrae (Phillips et al., 2013). It has been shown that when the vertebrae are fused, intervertebral motion between adjacent vertebrae is reduced, increasing the load on the surrounding tissues and adjacent IVD, which may further contribute to the development of IDD in adjacent segments (Kumar et al., 2001). For total disc replacement, prolonged wear of IVD prostheses prepared from metal and polymeric materials can lead to an immune inflammatory response and bone loss and are usually used only in the presence of single-segment IDD and in the absence of small joint disease. (Fritzell et al., 2001; Veruva et al., 2017; Werner et al., 2018). Surgical treatment can provide good relief to patients, but the high incidence of postoperative IVD stenosis and recurrence has become a major problem for physicians and patients (Scoville and Corkill, 1973; Hashimoto et al., 2019). Consequently, further explorations about more effective IDD treatment approaches are of great significance.

## 4 Regenerative therapy for IDD

In recent years, researchers have begun to investigate regenerative therapies aimed at regulating anabolic and catabolic metabolism within the IVD and restoring ECM and NPCs. These methods mainly include direct injection of growth factors, cell transplantation, gene therapy and tissue engineering.

In cell therapy, researchers transplant cells with differentiation potential into the IVD to help restore the number of cells in the disc, promote ECM synthesis, and immune regulation. For discs with different degrees of development and degeneration, the selection of the appropriate cell type and source is critical for successful cell transplantation and disc regeneration. Common cell types include MSCs, intervertebral disc-derived stem cells (IVDSCs), and pluripotent stem cells (PSCs) (Hwang et al., 2009). These cells are related in terms of embryonic origin and genealogy. Among them, MSCs lack low expression of the major histocompatibility complex II and co-stimulatory factors CD80, CD86 and CD40, which leads to their low immunogenicity (Fouillard et al., 2007; Jacobs et al., 2013). In addition, MSCs have the advantages of a wide source, high proliferative capacity, multispectral differentiation potential and low tumorigenicity (Tsaryk et al., 2017).This seems to offer a promising alternative to regenerative therapy. However, the microenvironment of IVD is characterized by hypoxia, nutrient deficiency, acidity, hyperosmolarity, and mechanical loading. Its internal microenvironment further deteriorates during IVD denaturation with mechanical overload and accumulation of inflammatory cytokines and proteases (Lyu et al., 2019; Ryu et al., 2020; Vasanthan et al., 2020; Guerrero et al., 2021). This causes the survival of cells injected into the disc to be a primary issue. Some studies have reported that leakage during MSCs injection therapy can further aggravate IDD (Vadalà et al., 2012). In addition, the potential ethical issues of cell therapy, how to obtain sufficient numbers of MSCs for clinical treatment and the associated rejection reactions are still issues that we need to address urgently.

Direct injection of growth factors into the IVD has also been used as a new treatment modality. Growth factors commonly used in injectable therapy include bone morphogenetic protein (BMP)-7, BMP-2, transforming growth factor-β (TGF-β), insulin like growth factor (IGF), epidermal growth factor (EGF) and differentiation factor (GDF)-5 etc., (Knezevic et al., 2017; Cecerska-Heryć et al., 2022). However, growth factors have a short half-life and often require multiple injections for treatment, which makes the risk of injection-related injury as well as infection significantly higher. Growth factors are peptides that target cells. However, for patients with advanced IDD, only a small number of cells are present within the IVD, which makes growth factor therapy for IDD difficult to implement clinically. Gene therapy mainly involves the transfer of target genes into IVD cells via viral or non-viral vectors, which are amplified *in vitro* and then subsequently injected into the IVD. These IVD cells with the target gene will be retained in the IVD for a long time, promoting IVD regeneration and improving the patient’s symptoms. (Sampara et al., 2018). However, this gene therapy is difficult to mitigate the complications associated with viruses used for gene transfection and non-viral vectors, and the clinical application of this method is still limited to medically life-threatening diseases (Han, 2020; Takeoka et al., 2020). Moreover, most of the relevant studies at this stage are still in the experimental stage and still need a lot of investment in research.

## 5 UCMSCs regenerate IVD

MSCs were first discovered and isolated in the bone marrow of rats (Friedenstein et al., 1966), and more and more organs and tissues have been reported to provide a stable source of MSCs, including bone marrow, adipose tissue, umbilical cord, pulp, placenta, skin, tonsils etc., (Zuk et al., 2001; Lai et al., 2014; Ryu et al., 2014; Yan et al., 2014; Miceli et al., 2021). MSCs from different sources have certain similarities in morphology, differentiation potential and immunophenotype, but there are still differences in population numbers, growth rates, colony frequencies, success rates of isolation, immunosuppressive ability and gene expression profiles (Thirumala et al., 2013).

### 5.1 Characteristics, sources, and treatment mechanisms of UCMSCs

Among them, BMMSCs and ADMSCs isolated from adult tissues are most frequently used for therapeutic purposes (Wei et al., 2014). However, as patients age, the number of bone marrow tissue and adipose tissue MSCs decreases, the difficulty of obtaining them and the associated risks of invasive operations are increased, and the ability to proliferate and differentiate decreases. In recent years, UCMSCs derived from allogeneic embryos have been used as an ideal alternative. Compared to MSCs derived from adult tissues, UCMSCs have the following advantages. In terms of access, UCMSCs derived from fetal discarded umbilical cords does not generate ethical controversy and without invasive manipulation, which allows for a large and stable source of UCMSCs(Lu et al., 2006; Fong et al., 2012). In terms of biological properties, UCMSCs exhibit higher proliferative and differentiation potential, and UCMSCs are three times more likely than BMMSCs to induce differentiation into chondrocytes and to produce collagen (Hsieh et al., 2010; Zhang et al., 2011). In terms of application prospects, the rate and stability of *in vitro* expansion of UCMSCs are also significantly higher than that of BMMSCs (Sarugaser et al., 2005; Vellasamy et al., 2012; Vawda and Fehlings, 2013), which allows UCMSCs to meet the large number of stem cells required for clinical use. Finally, in terms of safety, UCMSCs also have lower immunogenicity and cause fewer teratomas. However, at present, how to preserve cells for a long time, improve the stability of *in vitro* expansion, and whether the umbilical cord donor is healthy is an urgent problem for us to solve. The advantages and disadvantages of UCMSCs, BMMSCs, and ADMSCs are shown in Table 1.

**TABLE 1:** Common types of MSCs and their characteristics.

Cell sources	Advantages	Disadvantages
BMMSCs	High chondrogenic differentiation capacity	Invasive operation, high risk of infection, decreasing cell count with age
UCMSCs	More unlimited differentiation potential, lower immunogenicity, lower ethical risk	Donor shortage and potential donor health problems
ADMSCs	Rich tissue source, easy tissue collection and cell separation	Lower differentiation potential than BMMSCs

BMMSCs, Bone marrow mesenchymal stem cells; UCMSCs, Umbilical Cord Mesenchymal Stem Cells; ADMSCs, Adipose mesenchymal stem cells.

The human umbilical cord, the structure that connects the placenta to the developing fetus and thus provides the source of fetal nutrition, consists of two arteries and one vein surrounded by mucus connective tissue called the Wharton’s Jelly (WJ), with the outermost layer wrapped around the amniotic epithelium (Arutyunyan et al., 2016). The WJ makes up the majority of the cord and is composed of collagen fibrils, proteoglycans and stromal cells, which is the main source of UCMSCs. Among them, WJ-MSCs have significant advantages over MSCs from other parts of the umbilical cord in terms of quantity, ease of isolation, proliferation ability and viability.

At present, UCMSCs mainly achieves the purpose of treating IDD through the following main ways. On the one hand, UCMSCs have the potential to proliferate and multidirectionally differentiate, replenish NPCs by differentiating to NPCs, and promote the synthesis of ECM by NPCs (Steck et al., 2009; Fan et al., 2011). UCMSCs, on the other hand, have paracrine abilities. It can not only secrete growth factors and cytokines to regenerate IVD, but also secrete certain anti-inflammatory cytokines to regulate the inflammatory response of degenerative IVD, reduce pain and delay the process of IDD (Kuchroo et al., 2015; Shen et al., 2015; Beeravolu et al., 2018).

### 5.2 Studies related to UCMSCs for IVD regeneration

Although the number of reports on MSCs for the treatment of IDD is relatively large, most of these studies have focused on BMMSCs and ADMSCs. The existing *ex vivo* studies on UCMSCs mainly focus on the effects of UCMSCs on IVD, promoting the proliferation and differentiation of UCMSCs and improving their survival rate, while the related clinical studies need to be further explored. Zhang et al. (Zhang et al., 2015) injected UCMSCs isolated from human umbilical cords into canine IVD after labeling with EGFP, and found that UCMSCs survived in the IVD for a long time, delaying the rate of disc height decline as well as upregulating the expression of disc matrix genes, aggrecan, type II collagen (COL2), and SOX-9 [SRY (sex determining region Y)-box 9]. Wharton’s jelly cells (WJCs) still detectable in the IVD at 24 weeks. Perez-Cruet et al. (Perez-Cruet et al., 2019) found that by transplanting human UCMSCs into the IDD model of the rabbit, NPCs derivatives of MSCs expressed known NP-specific genes, SOX9, ACAN, COL2, FOXF1, and KRT19. Transplanted cells survived, dispersed, and integrated into the degenerated IVD. IVD augmented with NPCs showed significant improvement in the histology, cellularity, sulfated glycosaminoglycan and water contents of the NP. Ekram et al. (Ekram et al., 2021) showed that human UCMSCs can be differentiated into cartilage progenitor cells by using cartilage induction medium. Both chondroprogenitor cells and human UCMSCs in animal intervertebral discs express SOX9, transforming growth factor (TGF)-β1, ACAN, BMP2 and GDF5 genes as well as regulate inflammatory responses. Transplanted chondroprogenitors showed better survival, homing, and distribution in IVD as compared to normal MSCs. However, Wu et al. (Wu et al., 2017) found that NP progenitor cells isolated from degenerated IVDs exhibited lower proliferative and differentiation capacity compared to UCMSCs. This might account for the distinct NP microenvironment and the poor capacity for disc regeneration. Although UCMSCs can persist in the IVD for a long time and promote regeneration, microenvironmental and other factors may have some negative effects on their regenerative capacity.

To further aid in the differentiation of UCMSCs, Lee et al. (Chon et al., 2013) cultured UCMSCs in a hypoxic environment with three differentiation conditions: NP differentiation media (containing 2.5% Matrigel™ solution to provide for a pseudo-three-dimensional laminin culture system) with no serum, or the same media supplemented with either IGF-1 or TGF-β1. The study proved that a pseudo-three-dimensional culture condition (laminin-1 rich) promoted HUCMSC differentiation under no serum conditions. Neither growth factor treatment generated distinct differences in NP-like phenotype for HUCMSC as compared with no-serum conditions. Khalid et al. (Khalid et al., 2022) found that Sox-9 and Six-1 transcription factor-transfected UCMSCs greatly enhanced gene expression of TGF β-1, BMP, Sox-9, Six-1 and Aggrecan and accelerated differentiation to chondrocytes.

In addition to the ability of UCMSCs to differentiate directly into NPCs, there is also an effect on NPCs in degenerating IVD. Han et al. (Han et al., 2018), Ruan et al. (Ruan et al., 2012) and Zeng et al. (Zeng et al., 2020) cultured human WJCs with NPCs with and without direct cell-cell contact, respectively. The results showed that co-culture induced NP-like cell differentiation in WJCs, with significantly increased expression of NP-maker, OCT4, Nanog and Tie2 genes, and direct cell-to-cell contact, which could produce more favorable gene expression. Yuan et al. (Yuan et al., 2021) found that, human UCMSCs exosomes were found to effectively improve the viability of NP cells and protect them from pyroptosis through targeting METTL14.

Clinical trials of human UCMSCs make very few, and it is essential to investigate their effectiveness and safety. yang et al. (Pang et al., 2014) treated 2 patients with chronic discogenic low back pain with HUC-MSC transplantation. During the 2-year follow-up period, VAS and ODI scores decreased significantly. However, the reliability of this trial was poor due to the limited number of patients. In addition, some scholars have explored the direct injection of umbilical cord tissue into the affected area to relieve spinal disorders, and found significant pain relief in most patients through long-term follow-up, but there were individual cases with no significant pain relief, and no adverse events were reported (Buck, 2019; Ross et al., 2022).

Although UCMSCs show strong potential for the treatment of IDD, Matta et al. (Matta et al., 2021) found in their experiments that treatment with NTG-101 resulted in the suppression of inflammation induced p38 and NFκB, leading to the suppression of catabolic genes, but Smad-2/3, Erk-1/2 and Akt-dependent signaling activation, inducing anabolic genes for IVD-NP. A single injection of NTG-101 into the degenerating disc showed superior benefits compared to transplantation of human UCMSCs. A summary of trials related to the use of UCMSCs for regenerative treatment of IDD is shown in Table 2. These experiments demonstrated that UCMSCs can significantly delay or reverse IDD by promoting ECM synthesis, supplementing NPCs and affecting IVD endocytosis, etc. Compared with other stem cells, UCMSCs exhibit high proliferation and differentiation potential. We also demonstrated the feasibility of using UCMSCs in clinical practice.

**TABLE 2:** Studies related to UCMSCs for IVD regeneration.

References	Type of studies	Source of stem cells	Results
Ruan et al. (2012)	*In vitro*	Human UCMSCs + NPCs	The increases of relative gene expression in WJCs cocultured with cell-cell contact were larger than those cocultured without contact in all ratios
Chon et al. (2013)	*In vitro*	Human UCMSCs	HUCMSCs have the potential to differentiate into cells sharing features with immature NP cells in a laminin-rich culture environment
Pang et al. (2014)	Clinical Trials	Human UCMSCs	The pain and function improved immediately in the 2 patients. The VAS and ODI scores decreased obviously during a 2-year follow-up period
Zhang et al. (2015)	*In vivo*	Human UCMSCs + canine NP	WJCs can be present within the IVD for a long time and expression of matrix genes, aggregated glycans, type II collagen and SOX-9 are upregulated
Wu et al. (2017)	*In vitro*	Human UCMSCs + NPCs	NP progenitor cells isolated from degenerated IVDs exhibit lower proliferation and differentiation capacity compared to UCMSCs
Han et al. (2018)	*In vitro*	Human UCMSCs + NPCs	The genes such as aggregated glycan, type II collagen, and SOX-9 were highly expressed and direct cell-cell contact between WJCs and NPCs co-cultures produced more favorable
Perez-Cruet et al. (2019)	*In vivo*	Human UCMSCs + Rabbit NP	NP-specific gene upregulation with significant improvements in histology, cellularity, sulfated glycosaminoglycan and water content
Chon et al. (2013)	*In vitro*	Human UCMSCs + ECM	UCMSCs alleviate ECMD1 degradation via the p38 MAPK pathway
Ross et al. (2022)	Clinical Trials	Amniotic membrane and umbilical cord particulate	At 6 months, 75% of patients had relief of pain symptoms and no adverse events were reported
Zeng et al. (2020)	*In vitro*	Human UCMSCs + NPCs	CD29, CD105, OCT4, Nanog and Tie2 expression were increased and UCMSCs rejuvenated degenerated NP progenitor cells
Ekram et al. (2021)	*In vitro*	Human UCMSCs	Downregulation of pain and inflammation genes and *in vitro* induction of UCMSCs into chondroprogenitors lead to better regeneration of IVD
Yuan et al. (2021)	*In vitro*	Human UCMSCs + Human NP	UCMSCs exosomes stabilizes NLRP3 Mrna through the METTL14 pathway, thereby reducing IL-1β and IL-18 levels
Matta et al. (2021)	*In vivo*	Human UCMSCs + Rat IVD	Inflammation-induced inhibition of p38 and NFκB, Smad-2/3, Erk-1/2 and Akt-dependent signaling activation, and NTG-101 treatment were superior to UCMSCs
Khalid et al. (2022)	*In vivo*	Human UCMSCs + Rat IVD	Overexpression of Sox-9 and Six-1 greatly enhanced the gene expression of transforming growth factor beta-1 gene, BMP, Sox-9, Six-1, and Aggrecan, and protein expression of Sox-9 and Six-1
Buck (2019)	Clinical Trials	Amniotic membrane/umbilical cord	Pain score mean 4.9 to mean 3.5 at 4 weeks with no adverse effects

UCMSCs, Umbilical Cord Mesenchymal Stem Cells; NPCs, Nucleus pulposus cells; ECM, extracellular matrix; Rat, Rabbit; WJCs, Wharton’s jelly cells; VAS, visual analog scale; ODI, oswestry disability index; BMP, bone morphogenetic protein.

### 5.3 Tissue engineered of UCMSCs regenerative IVD

**FIGURE 1:**
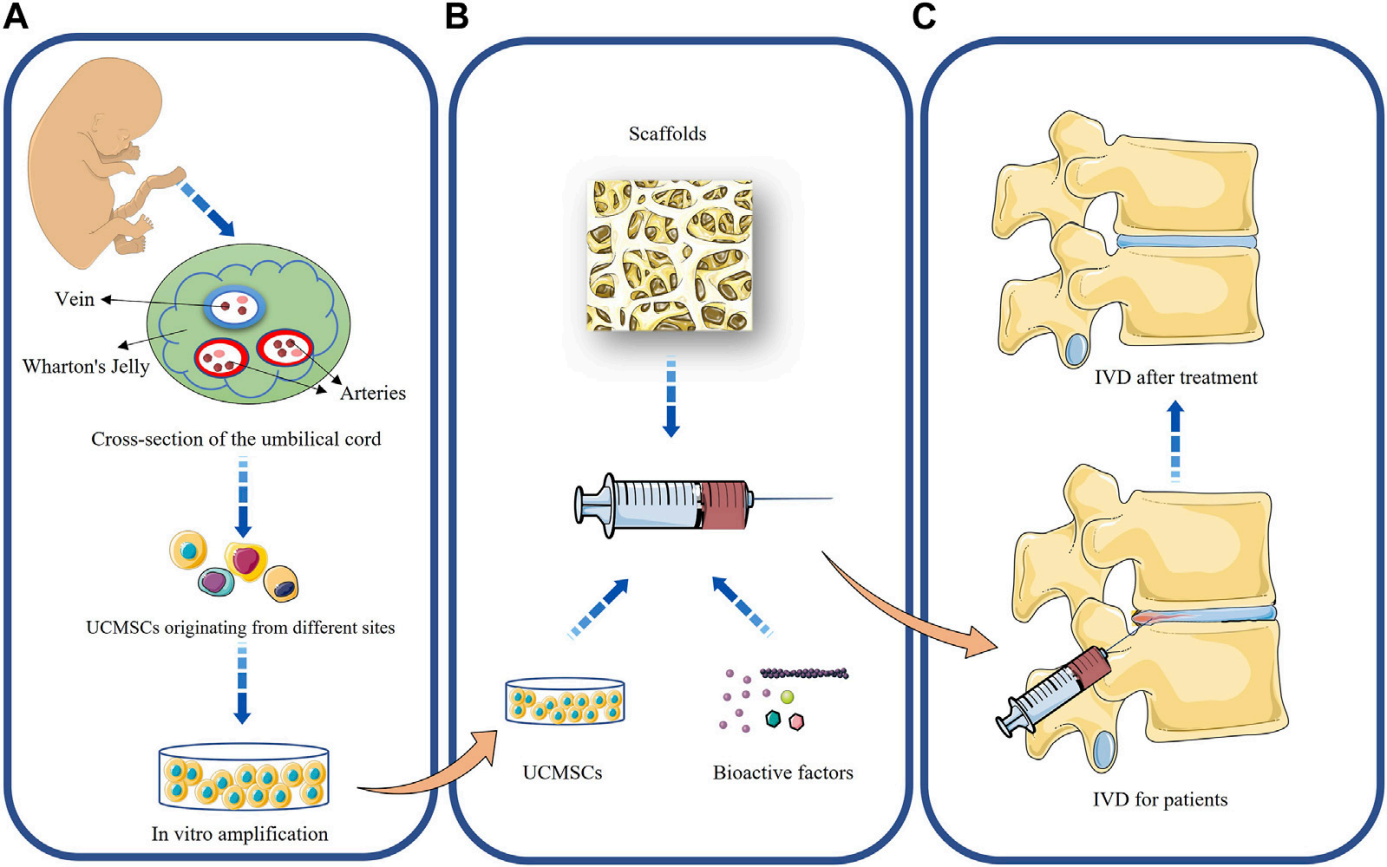
Application of UCMSCs to the treatment of intervertebral disc degeneration. **(A)**: Acquisition and *in vitro* amplification of UCMSCs; **(B)**: Tissue engineering of UCMSCs; **(C)**: Repair of intervertebral disc by UCMSCs.

Tissue engineering techniques have received increasingly widespread attention in order to eliminate the effects of the harsh microenvironment and mechanical loading within the IVD on the regenerative capacity of MSCs. Tissue engineering of the IVD consists of three key components: 1) seed cells that can regenerate IVD; 2) a biocompatible scaffold made of bioactive materials that can mimic ECM; 3) bioactive factors that promote cell proliferation and differentiation and migration (Gkantsinikoudis et al., 2022). A schematic diagram of the tissue engineering of UCMSCs is shown in Figure 1.

#### 5.3.1 Biomaterial scaffolds in tissue engineering

To help the survival of MSCs in the IVD, researchers have successively proposed culturing the cells *in vitro* under hypoxic conditions, heat treatment and the use of exogenous bioactive molecules (Kakudo et al., 2015; Baer et al., 2018; Chen et al., 2018). However, normal intra-IVD tissues consist of cells and ECM, and pretreatment for MSCs alone is not sufficient. With the development of biomaterials science in recent years, a variety of biomaterials have been used as scaffolds for cells to mimic the function of normal ECM. These biomaterials should be biocompatible and help restore cells and ECM within the IVD, reduce inflammatory response and inhibit pathological fibrosis (Huang et al., 2018). It is also indispensable to be able to stabilize the IVD under internal pressure load and to improve the stability of the spine. Biomaterial scaffolds are one of the three key elements in IVD tissue engineering, mostly hydrogel or solid scaffolds, which form the backbone of tissue regeneration. Differences in mechanical strength, void filling, cytotoxicity, immunogenicity, degradability and manufacturing cost exist among different materials, and it is particularly important to select the most appropriate type of scaffold material. According to the main source of materials, they can be classified as natural materials, synthetic materials and hybrid materials.

Reduced hydration within degenerated IVD is one of the main reasons for their development, and it is crucial to address the water content when selecting biomaterials for regenerating discs. Hydrophilic hydrogels are widely used in tissue engineering for IVD because they can be stored for longer periods of time and have a similar structure and function to ECM. Some scholars have verified that hydrogel scaffolds can help MSCs perform greater regenerative functions by mixing various materials into hydrogels and injecting them into animal IDD models in combination with BMMSCs (Smith et al., 2014; Zhang C. et al., 2021). However, it has also been reported that MSCs bound to fibrin carriers had little effect on porcine IVD regeneration and highly did not significantly improve (Acosta et al., 2011; Omlor et al., 2018).

#### 5.3.2 Studies related to tissue engineering of UCMSCs for IVD regeneration

Studies related to the use of UCMSCs alone for the treatment of IDD have yielded fairly promising results, but research in tissue engineering combining UCMSCs with biomaterial scaffolds still needs to receive more attention. To investigate the effects of tissue engineering of different types of biomaterials on UCMSCs and IVD, the investigators conducted relevant experiments.

Natural materials mainly include hydrogels, such as alginate, agarose, fibrin, hyaluronic, collagen, chitosan, and carboxymethylcellulose (Vaudreuil et al., 2019). These materials have excellent biocompatibility, better mimic natural ECM, and biodegrade to carbon dioxide and water *in vivo*. However, they have insufficient mechanical strength, rapid degradation rate, unstable biological properties and limited production capacity (Tang et al., 2021). Therefore, a single natural biomaterial scaffold is difficult to be used for tissue engineering of IVD. To investigate whether hydrogels prepared from natural materials are more useful for regenerating IVD in UCMSCs. Leckie et al. (Leckie et al., 2013) established a rabbit annulotomy model for IDD and subsequently randomized them into three groups, injected with UCMSCs alone, hydrogels alone and UCMSCs combined with fibrin hydrogels. Outcome analysis was performed by serial MRI observation as well as IVD histological staining at 4 weeks. The results showed that all three treatment groups exhibited a lower degree of degeneration than the control group in terms of total NP area and MRI index, with the UCMSCs combined with the hydrogel scaffold having the strongest regenerative effect on the IVD. And it showed significant fibrosis within the NP in the group injected with UCMSCs alone, and a significant decrease in the number of cells in the NP in the group injected with hydrogel alone, but maintained the highest number of ECM.

Choi et al. (Choi et al., 2020) found that T cells injected via needle resulted in cell damage and reduced viability and that cell viability was significantly increased when hyaluronan-methylcellulose (HAMC) was co-cultured with WJ-MSCs *in vitro*. This made the tissue engineering technique of combining HAMC with WJ-MSCs very attractive, so they randomly divided the rats into four groups to receive the following single intradiscal injections after establishing injury-induced IVD degeneration: 1) phosphate-buffered saline (PBS) vehicle, 2) HAMC, 3) WJ-MSCs, 4) WJ-MSCs/HAMC. The results showed that WJ-MSCs/HAMC had the strongest ability to regenerate IVD. Combined injection of WJ-MSCs and HAMC enhanced IVD regeneration through increase in cell survival, attenuation of the activation of iNOS, MMP-13, ADAMTS4 and COX-2, and significant upregulation of ECM, such as aggrecan and collagen type II. Ahn et al. (Ahn et al., 2015) found that the expression levels of TβRI/ALK5 and TβRII in WJ-MSCs from different donors differed between donors, which may affect the regenerative function of WJ-MSCs. They defined MSC-high TR as expression levels of TβRI/ALK5 and TβRII exceeding 5.0% and 20.0%, while MSC-low TR was defined at levels of approximately 1.0% and 7.0%. To this end, they injected cross-linked hyaluronic acid (XHA) scaffolds loaded with WJ-MSCs into a rabbit IDD model. The results showed that significant restoration of the disc water content in rabbits treated with MSC-highTR-loaded XHA scaffold in comparison to rabbits treated with the scaffold alone or MSC-lowTR-loaded XHA scaffold. In addition, morphological and histological analyses revealed that IVD regeneration was highest in rabbits transplanted with MSC-highTR-loaded XHA scaffold. The expression levels of TβRI/ALK5 and TβRII in WJ-MSCs could influence their secretion of cytokines such as GDF-15, MMP-1, and CCL-5 and their response to autocrine TGFβ ligands and that WJ-MSCs could improve IVD degeneration by releasing paracrine factors. Reppel et al. (Reppel et al., 2015) WJ-MSCs were embedded in alginate/hyaluronic acid hydrogels and then placed *in vitro* for culture. After 28 days of scaffold culture, results showed strong upregulation of cartilage-specific transcript expression. WJ-MSCs exhibited greater type II collagen synthesis than BMMSCs at both transcript and protein levels.

On the contrary, synthetic materials are relatively better than natural materials in terms of mechanical properties, while they are less biocompatible, hydrophilic and cell adhesion. In addition, degradation products of synthetic materials can induce inflammatory responses and reduce the rate of cell proliferation (Danhier et al., 2012). Synthetic materials mainly include poly (D, L-lactide) (PLA) and its derivatives, polyethylene glycol (PEG), polycarbonate urethane (PU), and poly (ε-caprolactone) (PCL). Considering the above reasons, composite materials combining natural and synthetic materials are widely used nowadays. It combines the advantages of both and reduces the impact of their respective disadvantages on regenerative IVD. Composite materials are a combination of two or more materials with different morphology or composition at the micro-/nanoscale (Tang et al., 2021). Li et al. (Li et al., 2017) established a novel biomimetic porous chitosan/poly (l-lactic acid) scaffold with human UCMSCs was applied in lumbar fusion. This approach demonstrated greater IVD regeneration than blank control and autologous bone in a rabbit model of IDD.

In addition, the decellularized matrix obtained by removing cells and antigens from autologous tissues through a series of methods also provides a new idea for tissue engineering of IVD (Xu et al., 2019; Qian et al., 2023). This type of scaffold is similar to ECM within IVD and can better assist cell adhesion, migration and proliferation. However, for humans, the stable access to sufficient amounts of decellularized matrix is the primary issue that limits its use for therapeutic purposes. Currently, WJ from the human umbilical cord is considered to be an ideal source of decellularized matrix scaffolds. These trials suggest that tissue engineering techniques have a stronger effect in restoring ECM and promoting cell proliferation and differentiation than UCMSCs injected alone for IDD. A summary of trials related to tissue engineering of biomaterial scaffolds combined with UCMSCs for regenerative treatment of IDD is shown in Table 3.

**TABLE 3:** Studies related to tissue engineering of UCMSCs for IVD regeneration.

References	Scaffolds	Results
Leckie et al. (2013)	Fibrin hydrogel	UCMSCs combined with hydrogels were superior to UCMSCs alone and hydrogels alone in terms of imaging performance, biomechanics, cell number and ECM content
Reppel et al. (2015)	Alginate/hyaluronic acid hydrogel	WJ-MSCs exhibited greater type II collagen synthesis than BM-MSCs at the transcript and protein levels
Ahn et al. (2015)	Hyaluronic acid	XHA hydrogel scaffold and high expression of TβRI/ALK5 and TβRII facilitate regeneration of IVD by WJ-MSCs
Li et al. (2017)	Porous chitosan/poly (l-lactic acid) scaffold	Tissue engineered for greater IVD regeneration than blank control and autologous bone
Choi et al. (2020)	Hyaluronan-methylcellulose hydrogels	Combined injection of WJ-MSCs and HAMC enhanced IVD regeneration through increase in cell survival, attenuation of the activation of iNOS, MMP-13, ADAMTS4 and COX-2, and significant upregulation of ECM.

UCMSCs, Umbilical Cord Mesenchymal Stem Cells; ECM, extracellular matrix; XHA, Cross-linked hyaluronic acid.

## 6 Summary and prospects

Currently, conventional treatment modalities for IDD provide good relief of symptoms in IDD patients, but their long-term outcomes are hardly satisfactory. A series of regenerative therapies have received much attention for their effectiveness in delaying or even reversing IDD. The higher proliferative differentiation potential, higher *in vitro* expansion rate and stability, lower immunogenicity and risk of infection make UCMSCs promising as an ideal choice for the treatment of IDD. *In vitro* and *in vivo* experiments have demonstrated that UCMSCs can differentiate into NPCs, promote ECM synthesis and regulate inflammatory responses within the IVD. However, the regenerative capacity of UCMSCs has reached opposite conclusions in some trials, and relevant clinical trials are scarce.The survival of UCMSCs under the harsh IVD microenvironment and pressure load is the main factor limiting their regenerative capacity. As the field of biomaterials continues to evolve, a new way of thinking is provided by the fact that UCMSCs have been shown to better perform their regenerative role by being attached to a variety of biomaterial scaffolds with excellent properties.

UCMSCs, as stem cells that have only recently entered the public eye, have been little studied, and existing experiments are similarly deficient. The main reason for this situation is the immaturity of UCMSCs in terms of extraction, storage and *in vitro* amplification. In recent years, cell banks for UCMSCs have been established in several countries with the aim of achieving access to large numbers of UCMSCs and helping heir clinical application. However, the number of established cell banks is currently much lower than expected. With the development and widespread use of 3D printing technology in the field of tissue engineering, 3D printed scaffolds may become a major trend. These scaffolds can be artificially adjusted to better meet the needs of UCMSCs survival and better repair the IVD. In addition, exosomes with lipid bilayers obtained by paracrine action of MSCs have been shown to promote tissue repair and regeneration. Because of their unique cell-free properties, they are significantly superior to MSCs in overcoming the microenvironment within IVD, immune rejection risk, tumorigenicity and ethical risk, which provides new ideas for further studies of UCMSCs. In the future, there are still many issues that need to be addressed for UCMSCs in regenerative treatment of IDD. More trials are needed to validate their effectiveness and safety in order to reduce the burden of IDD patients.
